# Persisting inter‐limb differences in patients following total hip arthroplasty four to five years after surgery? A preliminary cross‐sectional study

**DOI:** 10.1186/s12891-021-04099-7

**Published:** 2021-02-27

**Authors:** Stefanie John, David Weizel, Anna S. Heumann, Anja Fischer, Katja Orlowski, Kai-Uwe Mrkor, Jürgen Edelmann-Nusser, Kerstin Witte

**Affiliations:** 1 Sports Science, Faculty of Humanities, Otto-von-Guericke-University, Zschokkestraße 32, 39104 Magdeburg, Germany; 2 Department of Computer Science and Media, Brandenburg University of Applied Sciences, Magdeburger Straße 50, 14770 Brandenburg an der Havel, Germany

**Keywords:** Gait, Inter‐limb difference, Muscle strength, Range of motion, Balance, Total hip arthroplasty

## Abstract

**Background:**

Total hip arthroplasty (THA) is an effective procedure for patients with end-stage hip osteoarthritis. However, whether or not pre-operatively existing functional deficits are persisting several years post-surgery in the affected limb has not been thoroughly researched. Therefore, the primary aim of this preliminary study was to include patients four to five years after undergoing THA and to investigate potential differences between the operated and non-operated leg in hip strength, range of motion (ROM), balance, and gait. The secondary aim was to compare these values from the operated leg of the patients to those of the legs of healthy subjects.

**Methods:**

Sixteen patients (age: 65.20 ± 5.32 years) following unilateral THA (post-operation time: 4.7 ± 0.7 years) and ten, healthy, age-matched control subjects (age: 60.85 ± 7.57 years) were examined for maximum isometric hip muscle strength, active ROM of the hip joint, balance and gait on both limbs. Paired t-tests were used to assess the inter-limb differences in the THA group. Analyses of covariance (ANCOVA) were performed to compare groups, using age as a covariate.

**Results:**

The analysis of inter-limb differences in patients following THA revealed significant deficits on the operated side for hip abduction strength (*p* = 0.02), for hip flexion ROM (*p* < 0.01) and for balance in terms of the length of center of pressure (COP) (*p* = 0.04). Compared to values of the control subjects, the patients demonstrated significantly reduced hip strength in flexion, extension and abduction (*p* < 0.05) on the operated leg as well as reduced ROM measures in hip flexion, extension and abduction (*p* < 0.05).

**Conclusions:**

The first results of this explorative study indicated that inter-limb differences as well as reduced hip strength and hip ROM compared with control subjects were still present four to five years after THA. These persisting asymmetries and deficits in patients following THA may be one explanation for the decrease in health-related quality of life (HRQoL) seen in patients over the years after surgery. Further studies are required to replicate these findings with a larger sample size.

**Trial registration:**

DRKS, DRKS00016945. Registered 12 March 2019 – Retrospectively registered,

## Background

Hip osteoarthritis is one of the most frequent joint diseases, which develops in approximately one in four people over their lifetime [1]. Patients with hip osteoarthritis suffer from pain, reduced muscle strength, function and balance as well as limited range of motion (ROM) in the affected hip joint [2, 3]. First-line treatment of these impairments consists of conservative therapeutic interventions such as exercise therapy and physiotherapist-led treatments. However, if these treatment methods are ineffective and fail to provide improvements, total hip arthroplasty (THA) is required. Following THA, patients reported significant improvements for pain and hip function [4, 5]. One year after the implantation of the artificial hip joint, the overall patient satisfaction with the operation result is high, ranging between 88 % [6] and 93 % [7]. However, several studies have shown that from 12 months post-THA onwards, the patients’ reported health-related quality of life (HRQoL) started to decrease over time [8, 9], especially due to a decline in physical function [10]. Comparing the physical function of patients to healthy, age-matched control subjects, deficits in muscle strength, balance and gait were observed one to two years after the surgery [11–13]. The impairment of physical function and decreasing HRQoL have been associated with musculoskeletal comorbidities for patients after THA [14]. Seven years post THA, one third of the patients suffered from low back pain and half with general musculoskeletal pain [10]. Musculoskeletal pain can be induced by muscle asymmetries and muscle imbalances [15]. In the first year after THA, asymmetries between the operated and non-operated side were found for the muscle strength of the lower extremities [16]. Two years after THA, significant between-limb differences were still detected for the maximum isometric strength of the hip muscles [17]. Concerning static standing, walking and sit-to-stand transition tasks, asymmetric limb loading was also demonstrated 1.5 years after unilateral hip replacement [18].

However, most studies only monitored differences between the operated leg and the non-operated one for a follow-up period of one to two years. Data on inter-limb differences beyond two years post-THA are rare. Examining potential asymmetries beyond the two years is important as asymmetric limb relations and asymmetric joint loading may lead to overloading the non-operated limb inducing an early development or accelerated progression of osteoarthritis of the non-operated limb [18, 19].

Therefore, the primary aim of the study was to include patients who had undergone THA four to five years ago and to investigate potential inter-limb differences in muscle strength of the hip, hip ROM, balance, and spatiotemporal gait parameters. The secondary aim was to compare the values of the operated leg of patients following THA to values of control subjects. This study had the purpose of an exploratory investigation in order to detect if inter-limb differences or deficits were still present at all in patients years after the surgery. We hypothesized that patients would show between-limb asymmetry in hip strength, hip ROM, balance and gait parameters four to five years after THA. Secondly, we hypothesized that the values of the operated leg would show deficits when compared to values of healthy, age-matched peers.

## Methods

### Participants

 Participants for the THA group and the control group were recruited through advertisements and articles in local newspapers. The THA group comprised patients who had undergone unilateral THA four to five years ago. Further inclusion criteria were an age between 50 and 70 years as well as being physically active at least two times a week. Healthy, age-matched and physically active participants were included in the control group. Exclusion criteria were neurological or cardiovascular diseases and acute injuries of the musculoskeletal system. Healthy controls were also excluded if they had diagnosed osteoarthritis in lower extremity joints. All participants gave written consent to participate in this study after being informed about the procedure, its purpose and possible risks linked to the participation. The study was approved by the local ethics committee of the Otto-von-Guericke-University Magdeburg presided by Dr. med. Norbert Beck and carried out in line with the Declaration of Helsinki (no. of vote: 155/18 on December 3, 2018). It was retrospectively registered in the German Registry of Clinical Trials under the ID: DRKS00016945.

### Measurement protocol

For this cross-sectional study, data collection was carried out between January 2019 and June 2019. The participants were asked to attend one testing, in which all measurements were conducted. The measurements consisted of examinations of isometric strength of the hip muscles, hip ROM, balance and gait. In the THA group and the control group, data were collected on both legs. Regarding the THA group, the legs were differentiated in the operated and the non-operated side.

### Maximum isometric hip strength analysis and active hip ROM analysis

**Fig. 1:**
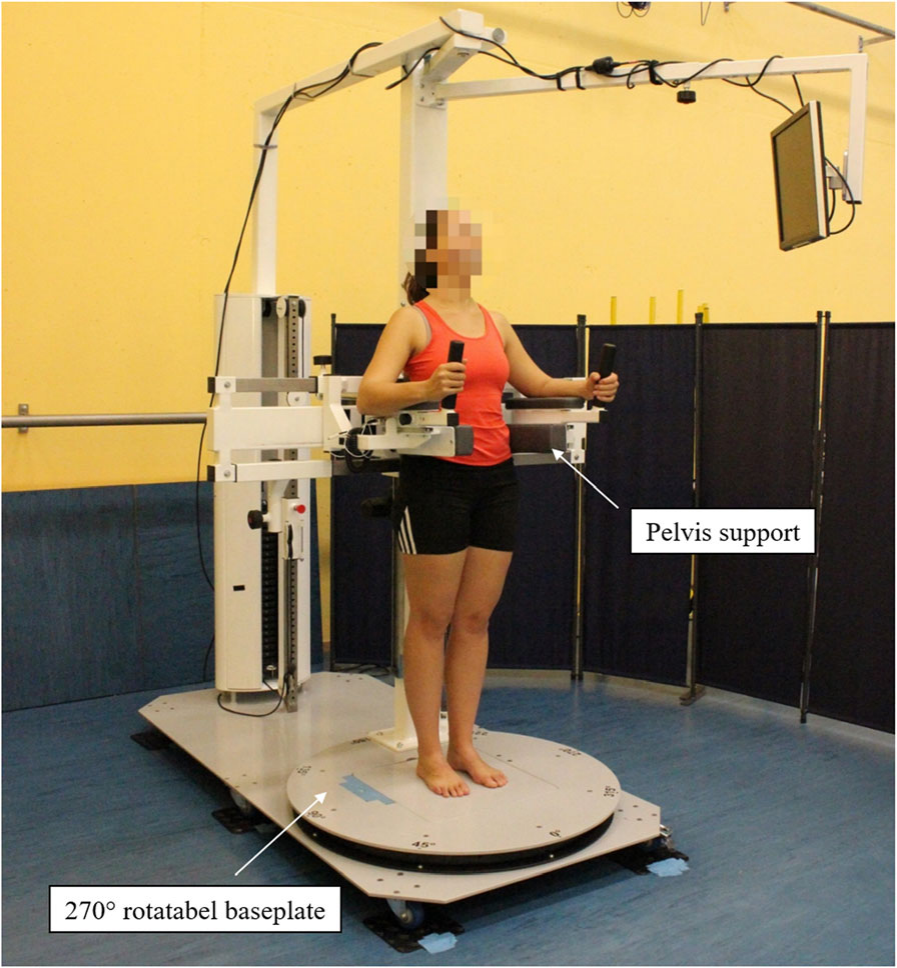
Overall display of the diagnostic machine

The examinations of the isometric strength of the hip muscles and of the active hip ROM were performed in a self-developed diagnostic machine (Fig. 1). The pelvis support helps the patients to maintain a fixed position and to avoid compensational movement during the measurements. The diagnostic machine contains a 270° rotatable baseplate, which enables the strength measurement of patients in different directions while the patients can just remain in their position.

The reliability of the isometric hip strength measurement as well as of the hip ROM measurement of the diagnostic machine had been investigated before the examinations. 24 healthy individuals participated in the reliability studies (Study 1 (hip ROM): *n* = 10, 28.4 ± 5.7 years; Study 2 (hip strength): *n* = 14, 21.3 ± 2.1 years). Reliability of the hip strength and ROM measurement were examined in a test-retest design with seven days between measurements. To examine the test-retest reliability, intra-class correlation coefficients (ICCs) were calculated based on a single-rating, absolute agreement and a two-way mixed effects model [20]. The ICCs showed values ranging between 0.85 and 0.95 for the isometric hip strength measurement (hip flexion, extension, abduction, and adduction) and values ranging from 0.65 to 0.84 for the hip ROM measurement (hip flexion, extension and abduction). According to Koo and Li, these ICCs indicate good to excellent reliability [20] with one exception for the measurement of hip flexion (ICC = 0.65), which is interpreted as moderate reliability. The results suggest that the diagnostic machine provides an environment to reliably quantify maximum isometric hip strength and active hip ROM.

**Fig. 2:**
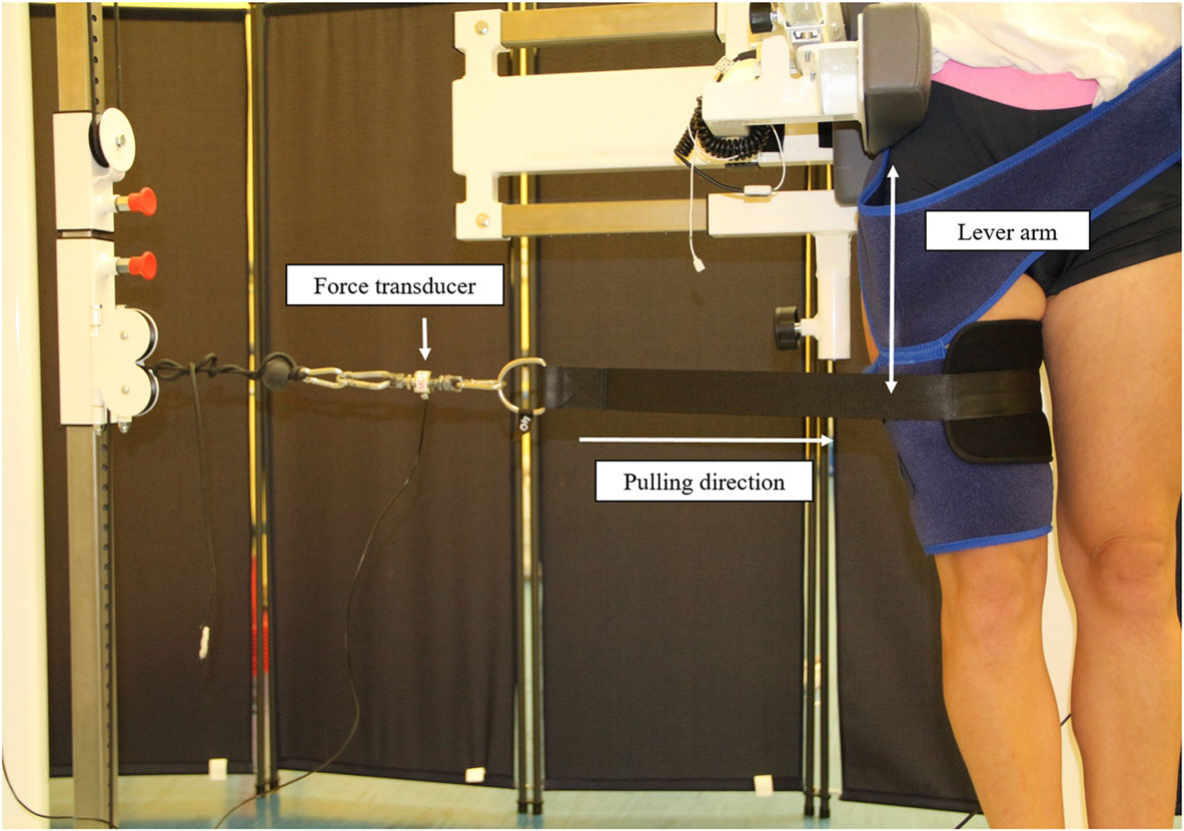
Measurement set-up for quantifying maximum isometric strength for hip adduction

For the examination of the maximum isometric strength of the hip muscles, subjects were instructed to stand in an upright position in the diagnostic machine. The pelvis support helped the participants to remain in this position. A neoprene brace was placed distally at the thigh as an attachment possibility for the hauling rope. An integrated force transducer (Hottinger Baldwin Messtechnik GmbH, Darmstadt, Germany) measured the isometric strength in the respective pulling directions hip flexion, extension, abduction and adduction in the neutral hip position (Fig. 2).

For each motion direction, one pretest and two main tests were performed. Subjects were instructed to build up strength and contract maximally without an abrupt push. A resting period of one minute between each trial was maintained. Force data from the strength analysis were acquired at 1000 Hz and filtered in Matlab with a 4th order Butterworth low-pass filter (5 Hz). Out of the two main trials, the trial with the highest torque was normalized to the body mass of the participants and used for further analyses. The distance between the greater trochanter and the point of applied force (the middle of the neoprene brace) served as the lever arm (Fig. 2).

**Fig. 3:**
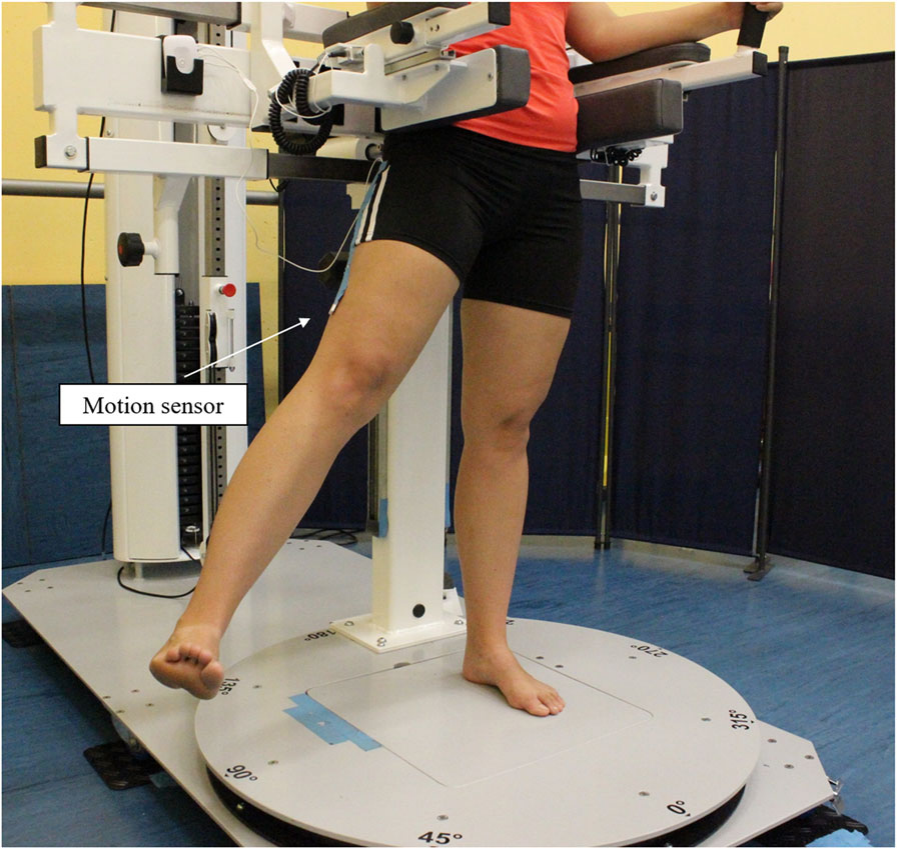
Measurement of active hip abduction

For the examination of the hip ROM, subjects were also standing in the diagnostic machine fixated right above the pelvis in order to avoid compensational movements with the upper body but still providing free movement of the hip joint. Active ROM of the hip was measured in flexion, extension and abduction in a standing position. Adduction was excluded due to potential risk of luxation of the prosthesis. The angles of the three movement directions were quantified with an acceleration sensor (PLUX-Wireless Biosignals S.A, Lisbon, Portugal) placed distally on the lateral side of the thigh. After initializing the sensor in the neutral zero position, participants were instructed to slowly perform three maximal hip flexion movements followed by three maximal extension and abduction movements (Fig. 3). Particular attention was paid to the participants to not modify their upper body position and to cleanly execute the motion (hip abduction, flexion, extension) in the respective motion axis. Data from the motion analysis were acquired at 1000 Hz and filtered in Matlab with a 4th order Butterworth low-pass filter (5 Hz). Out of the three trials, the maximum hip angles in flexion, extension and adduction on each side were extracted for further analyses.

### Balance assessment

Static balance was assessed in the bipedal and single-leg stance using a force plate (PLUX-Wireless Biosignals S.A, Lisbon, Portugal). For the bipedal stance, subjects were asked to take off shoes and stand with both legs, hip width apart, on the force plate with the arms hanging down at the sides. Two trials with a duration of thirty seconds were recorded. For the single-leg stance, the participants were instructed to position one leg in the center of the force plate, slightly lifting off the other foot and fixating the wall in front of them. Before collecting data, the participants were asked to practice this posture. Two trials on each leg were captured with a duration of ten seconds. The acquisition time in the single-leg stance was limited to ten seconds as most subjects were not able to hold the position for much longer. Balance data were sampled at 250 Hz and further processed using Matlab (Version 2018b, The Math-Works Inc., Natick, MA). The dataset was filtered applying a 4th order Butterworth low-pass filter with a 10 Hz cut-off frequency. The total length of the center of pressure (COP) during bipedal and single-leg stance was computed as well as the standard deviations (SD) of the COPx and COPy for mediolateral (ML) and anteroposterior (AP) directions [21]. The best trials of each leg were chosen for further analyses.

### Gait analysis

The gait analysis was performed with InvestiGAIT, an inertial sensor-based system consisting of four Shimmer3 sensors (Shimmer, Realtime Technologies Ltd, Dublin, Ireland) and an in-house Matlab program for recording and analyzing gait data. Two of the inertial sensors were laterally placed above each ankle. In order to quantify the movement of the hip and the upper body, the third and the fourth Shimmer sensors were centered at the height of the posterior superior iliac spine and at the thoracic vertebra II [22]. The subjects were asked to walk a predefined distance (12.5 m) marked by two pylons at their self-selected, comfortable walking speed. For each participant, twelve gait sequences were recorded. Outcome spatiotemporal gait parameters involved step length, stance and swing duration as well as one-leg-stance as a percentage of the gait cycle. These parameters can also be used for inter-limb examinations as they are calculated for both legs (affected/non-affected) and therefore provide information about gait symmetry or asymmetry [23, 24].

The gait parameters of the InvestiGAIT system are calculated based on the identification of gait events including initial contact (IC), midswing point and terminal contact (TC). These events are detected as local minima (IC, TC) or local maxima (midswing) in the signals of the z-axis of the ankle gyroscopes, which describe the angular velocities of the shanks in the sagittal plane. More detailed information about the detection of gait events and calculation of the gait parameters of the InvestiGAIT system are provided in Orlowski and Loose [25] and Orlowski et al. [26]. The InvestiGAIT system has been confirmed to be a valid and reliable system to investigate human gait in a clinical setting [22, 26].

### Statistical analyses

All analyses were performed using SPSS 25 (SPSS Inc., Chicago, IL) with a significance level set to *p* < 0.05. The data were tested for normal distribution applying the Shapiro-Wilk test. To investigate potential differences between the operated and non-operated side of the THA group, a paired t-test was applied for each parameter. In case of violation of normal distribution, the nonparametric Wilcoxon test was used. For group comparisons, the demographic variables of the THA group and the control group were verified for significant differences applying the unpaired t-test, Mann-Whitney U and Chi-squared test. As an age difference- although not significant- was observed, analyses of covariance (ANCOVA) were applied to assess group differences in each parameter, using age as a covariate. The data of the operated side of the THA group were compared to the averaged data of the right and left leg of the control group. For intrasubjective comparisons, effect sizes were calculated using Cohen’s d_z_ for within-subjects designs [27]. In case of non-normally distributed data, the effect sizes were determined with the correlation coefficient r. For group comparisons, effect sizes were calculated applying Cohen’s d_s_ for in between-subjects designs [27]. Values for d = 0.2 were interpreted as a small, d = 0.5 as a medium and d = 0.8 as a large effect. Effect sizes for r were interpreted as small (*r* = 0.1), medium (*r* = 0.3) and large (*r* = 0.5) [28].

## Results

### Participants

Sixteen patients with unilateral hip replacement (10 females, 6 males) voluntarily participated in this study. Post-operation time amounted to 4.7 ± 0.7 years and the operation side was seven times the right and nine times the left hip. Further subject characteristics are presented in Table 1. Ten healthy, age-matched subjects served as controls (6 females, 4 males). No significant differences in terms of age, sex distribution, height and weight were observed between groups (Table 1).

**Table 1:** Demographic characteristics of THA group and control group

	THA group (*n* = 16)	Control group (*n* = 10)	*p*-value
Age [years]	65.20 ± 5.32	60.85 ± 7.57	0.10
Female [%]	62.50	60.0	0.90^#^
Height [m]	1.67 ± 0.10	1.72 ± 0.13	0.28
Mass [kg]	72.28 ± 17.41	71.10 ± 20.45	0.52^U^
Post-Op time [years]	4.66 ± 0.72	-	-

^#^ Chi-squared test

^U^ Mann-Whitney-U-Test

### THA group: operated vs. non‐operated side

Comparing the parameters of the operated hip with the non-operated side, three significant differences were detected (Table 2). The maximum isometric strength analysis revealed a significant deficit in hip abduction on the operated side with a medium effect size (*p* = 0.02, d = 0.66). Concerning the motion analysis, a significant deficit on the operated side was evident for hip flexion with a large effect size (*p* < 0.01, d = 1.09). Regarding the balance analysis, the COP length of the single-leg stance was significantly longer on the operated side than on the non-operated one with a medium effect size (*p* = 0.04, d = 0.56). No significant inter-limb differences were found in the spatiotemporal gait parameters (*p* > 0.05).

**Table 2:** Balance, motion, strength and gait parameters of the operated and non-operated side of patients following THA

	Operated side	Non-operated side	*p*-value	Cohen’s d
Strength [Nm/kg]				
Flexion	1.43 ± 0.35	1.46 ± 0.30	0.67	0.11
Extension	0.83 *±* 0.17	0.86 *±* 0.20	0.51	0.17
Abduction	0.96 *±* 0.23	1.06 *±* 0.28	0.02*	0.66
Adduction	1.05 *±* 0.29	1.06 *±* 0.29	0.86	0.05
Motion [°]				
Flexion	89.13 ± 17.27	100.19 ± 12.24	0.001*	1.09
Extension	33.92 *±* 7.22	33.04 *±* 8.79	0.44^w^	0.19^r^
Abduction	36.74 *±* 8.58	37.65 *±* 10.45	0.79	0.07
Balance [mm]				
COP length	783.1 ± 309.65	696.8 ± 293.33	0.04*	0.56
SD in AP	9.23 ± 4.17	9.42 ± 5.30	0.96^w^	0.01^r^
SD in ML	10.01 ± 5.17	7.87 ± 2.74	0.09^w^	0.24^r^
Gait				
Step length [m]	0.74 ± 0.09	0.73 ± 0.09	0.87	0.04
Stance duration [s]	0.58 *±* 0.08	0.58 *±* 0.08	0.94	0.02
Swing duration [s]	0.47 *±* 0.04	0.47 *±* 0.03	0.98	0.01
One leg stance [%]	44.90 *±* 2.57	44.94 *±* 3.34	0.96	0.01

^w^ Wilcoxon-test

^r^ Correlation coefficient r

* Differences statistically significant (p < 0.05)

### THA group vs. control group

For group comparisons, the values of the operated leg of the patients following THA were compared to the averaged values (right and left leg) of the control subjects (Table 3).

The examination of the maximum isometric hip strength showed strength deficits of the operated legs of the patients following THA compared to the legs of the control subjects. Significantly reduced strength values were observed for hip flexion (*p* = 0.01), hip extension (*p* < 0.01) and hip abduction (p < 0.01) with large effect sizes (d > 0.8). The motion analysis revealed significant group differences for all motion directions with lower motion angles on the part of the THA group (*p* < 0.05). Large effect sizes were seen for hip flexion (d = 1.31), hip extension (d = 0.89) and hip abduction (d = 2.30). No significant group differences were detected in the balance parameters or in the gait parameters (*p* > 0.05).

**Table 3:** Comparison of balance, motion, strength, and gait parameters between THA group and control group

	THA group	Control group	*p*-value	Cohen’s d
Strength [Nm/kg]				
Flexion	1.43 ± 0.35	1.90 ± 0.40	0.01*	1.09
Extension	0.83 *±* 0.17	1.18 *±* 0.23	0.001*	1.56
Abduction	0.96 *±* 0.23	1.41 ± 0.24	< 0.001*	1.74
Adduction	1.05 *±* 0.29	1.29 *±* 0.23	0.08	0.75
Motion [°]				
Flexion	89.13 ± 17.27	107.07 ± 6.79	0.004*	1.31
Extension	33.92 *±* 7.22	39.85 *±* 8.75	0.04*	0.89
Abduction	36.74 *±* 8.58	52.19 *±* 5.13	0.000*	2.30
Balance bipedal stance [mm]				
COP length	417.06 ± 162.28	382.81 ± 121.39	0.63	0.20
SD in AP	3.18 ± 1.00	2.84 ± 0.94	0.74	0.14
SD in ML	5.31 ± 1.55	4.19 ± 1.09	0.06	0.81
Balance single-leg stance [mm]				
COP length	783.10 ± 309.65	676.63 ± 202.43	0.63	0.20
SD in AP	9.23 ± 4.17	7.41 ± 1.49	0.37	0.37
SD in ML	10.01 ± 5.17	7.83 ± 1.25	0.29	0.44
Gait				
Velocity [m/s]	1.29 ± 0.25	1.43 ± 0.17	0.10	0.68
Step length [m]	0.74 ± 0.09	0.80 ± 0.08	0.14	0.61
Stance duration [s]	0.58 *±* 0.08	0.56 *±* 0.07	0.37	0.37
Swing duration d [s]	0.47 *±* 0.04	0.47 *±* 0.03	0.68	0.17
One leg stance [%]	44.90 *±* 2.57	45.39 *±* 2.56	0.26	0.47

* Differences statistically significant (*p* < 0.05)

## Discussion

Most studies have focused on investigating the clinical and functional outcome of patients following THA up to two years post-surgery. This study had the primary goal to include patients, who had undergone THA four to five years ago, and to investigate potential differences between the operated and non-operated side. Persisting deficits on side of the operated leg were found for single parameters in hip muscle strength, hip ROM and balance. In comparison with values of healthy subjects, the patients following THA demonstrated reduced hip muscle strength and hip ROM.

The isometric maximum strength analysis revealed that hip strength values were reduced on the operated side, but only the hip abductors demonstrated a significant inter-limb difference with an average deficit of 0.10 Nm/kg (9 %) on part of the operated side. Similar to our results, Rasch et al. showed that a significant strength deficit of the hip abductors (15 %) remained on the operated side two years after THA whereas the pre-operatively existing significant inter-limb strength asymmetries in hip extension, hip adduction and hip flexion had recovered within the two years [17]. The strength difference of 9 % between the operated and the non-operated side seen in our study does not seem so high when comparing it to lower-limb strength asymmetries of 10 % reported for young asymptomatic healthy humans [29]. However, regarding the age of the patients and the affected muscle group, this inter-limb difference might be clinical relevant. Hip abductors are known to be important for stabilizing pelvis during ambulation and unipedal tasks [30]. Unilateral weakness of hip abductors has been shown to influence gait and balance [30, 31] and therefore may affect many tasks of the everyday life. When comparing the hip strength values of the operated side to the values of control subjects, a general weakness of the hip muscles were detected for the patients following THA. Significant strength differences were seen for hip flexion, hip extension and hip abduction. Similar results were reported in the study of Bertocci et al. [32]. A general weakness of the hip muscles, especially of the hip abductors, has been associated with poorer physical function [33] and low back pain [34]. Concerning the strength analysis, this study revealed persisting inter-limb asymmetry for the hip abductors as well as a persisting general hip strength deficit for patients four to five years after THA. This may partly explain the patients’ reported increasing impairment of physical function [10] and decreasing HRQoL over the years [8].

Concerning the inter-limb examination of balance parameters in this study, a significantly increased COP length on the operated side in the single-leg stance was observed for the patients following THA. In previous studies, increased COP variables were seen as increased body sway and interpreted as a decreased performance of the postural system [35, 36]. Trudelle-Jackson et al. also investigated inter-limb differences in the single-leg stance in patients following THA and showed significant lower measures of postural stability on the side of the operated hip one year after the surgery [37]. The increased COP sway on the operated side seen in this study may be due to the detected abductor weakness on the operated leg of the patients following THA. It may have been harder for the patients to stabilize the pelvis in the horizontal plane on this leg causing greater sway. This can also be seen in the trend of a greater mediolateral displacement of the COP on the operated side when compared to the non-operated one. Besides influencing postural control, unilateral hip abductor weakness has been shown to affect gait pattern. In our study, no inter-limb differences between the operated and non-operated leg were detected for the spatiotemporal gait parameters implying symmetric gait for patients four years post-THA. This is in line with most studies, which demonstrated a recovery of asymmetric gait of patients following THA within one to two years [17, 24].

The active ROM analysis of the hip joint, however, revealed a significant inter-limb difference for patients following THA. Patients showed a significantly reduced hip flexion angle on the operated side with an average deficit of 11° compared to the non-operated one. Similar results were obtained in the study of Häkkinen et al. One year after hip resurfacing the patients showed a 6° lower flexion angle on the side of the operated hip [38]. When comparing the hip flexion angle of the operated side to controls, the deficit in hip flexion on part of the patients following THA became more evident. A significant difference of 18° were observed for hip flexion between groups. Significantly reduced hip angles were also detected for hip extension and hip abduction on part of the patients following THA. Restoring hip ROM is just as important as restoring hip strength for the patients following THA as low ROMs were associated with high levels of disability [39]. Inter-limb differences in lower-limb joint ROM and strength may also lead to asymmetric joint loading which may result in the development of disorders in contralateral and adjacent joints. Therefore, symmetric inter-limb relations of muscle strength and ROM should always be pursued in order to prevent overloading one side.

This study showed that four to five years after THA, significant asymmetries between the operated and the non-operated leg were still present for single parameters partly confirming our hypothesis on persisting inter-limb differences years after THA. Compared to the values of control subjects, significantly reduced values for hip strength and hip ROM were found for the operated leg. These findings confirm our hypothesis on persisting deficits of the operated leg years after THA.

Some limitations have to be addressed in our study. First, no data were collected on the operation method. Different operation approaches may be associated with different muscle and tissue damages [40], which might have had an influence on the results of our isometric maximum strength analysis. In future studies, patients following THA should also be controlled for osteoarthritis in other joints as this could affect the isometric strength as well. The short data acquisition time in the single leg stance also needs to be mentioned as a limiting factor. According to Scoppa et al. [41], collection time for COP-related balance data should not be less than 25 seconds. As participants were only capable of holding the single leg stance for a short time, alternative test conditions for measuring inter-limb differences in balance should be considered.

Last, the small sample size of patients following THA is the major limitation of this study. This study had more of an exploratory character to detect if any inter-limb differences were still present at all in patients years after the surgery. As this study indicated persisting asymmetries and deficits, studies with larger numbers of participants should be conducted to confirm the significance of these results.

## Conclusions

Four to five years after THA, asymmetries between the operated and the non-operated leg were still present. Significant deficits were revealed on the operated side for hip abduction strength, hip flexion ROM and for the COP length in the single-leg stance. Especially the persisting strength asymmetry of the hip abductors may be clinically relevant due to their important function in stabilizing trunk and pelvis. Compared to values of healthy control subjects, the operated leg of the patients following THA showed significantly reduced hip strength and hip ROM values. The detected inter-limb asymmetries as well as the observed persisting strength and ROM deficits in this study may serve as an explanation for the increase in musculoskeletal pain and decreasing quality of life seen in patients over the years after THA. Therefore, postoperative training should be continued for months to years after the surgery targeting all hip muscles and hip ROMs in order to reduce asymmetries and deficits.

## Data Availability

The datasets used and/or analyzed during the current study are available from the corresponding author on reasonable request.

## Abbreviations

THA: Total hip arthroplasty
ROM: Range of motion
COP: Center of pressure
ANCOVA: Analysis of covariance
HRQoL: Health-related quality of life
IC: Initial contact
TC: Terminal contact
SD: Standard deviation
AP: Anteroposterior
ML: Mediolateral
ICC: Intra-class correlation coefficient
